# Characterization of a Photodiode Coupled with a Si Nanowire-FET on a Plastic Substrate

**DOI:** 10.3390/s101009118

**Published:** 2010-10-12

**Authors:** Kiyeol Kwak, Kyoungah Cho, Sangsig Kim

**Affiliations:** 1 Department of Electrical Engineering, Korea University, Seoul 136-713, Korea; E-Mails: kky0819@korea.ac.kr (K.K.); chochem@korea.ac.kr (K.C.); 2 Department of Nano Semiconductor Engineering, Korea University, Seoul 136-713, Korea

**Keywords:** *pn* heterojunction photodiode, FET, optical sensor, plastic substrate, nanowire, 81.05.Lg, 78.67.-n, 73.40.Lq, 78.67.Uh, 85.30.Kk

## Abstract

In this study, a laterally coupled device composed of a photodiode and a Si nanowires-field-effect transistor (NWs-FET) is constructed on a plastic substrate and the coupled device is characterized. The photodiode is made of *p*-type Si NWs and an *n*-type ZnO film. The Si NWs-FET is connected electrically to the photodiode in order to enhance the latter’s photocurrent efficiency by adjusting the gate voltage of the FET. When the FET is switched on by biasing a gate voltage of −9 V, the photocurrent efficiency of the photodiode is three times higher than that when the FET is switched off by biasing a gate voltage of 0 V.

## 1. Introduction

Inorganic nanomaterial-based plastic optoelectronics is a rapidly developing field, since inorganic nanomaterials can be easily fabricated on plastic substrates by spin-coating [1–4]. Recently, the photolithography-based fabrication and direct transfer process of semiconducting nanowires (NWs) from a wafer has begun to emerge as a promising technology for the fabrication of such inorganic NWs on plastic substrates [5–7]. In actual practice, field-effect transistors (FETs) with channels composed of Si NWs transferred onto plastic substrates have revealed their usefulness as a functional component for flexible electronics [8–10]. Nevertheless, as yet the transferred NWs have not been used as a material for plastic optoelectronics. Therefore, in this study, we investigate the possibility of using the transferred Si NWs as an active component for optoelectronics on plastic substrates.

**Figure 1.**
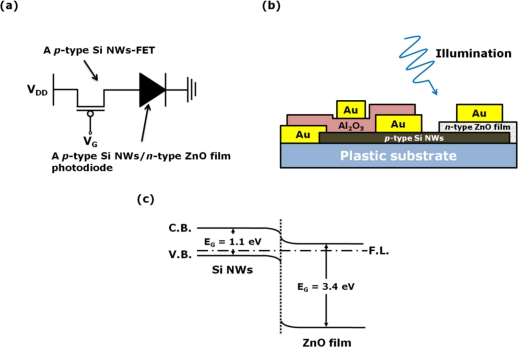
**(a)** Electronic circuit diagram; **(b)** cross-sectional schematic; and **(c)** schematic energy band diagram of a laterally coupled device composed of a *p*-type Si NWs-FET and a *p*-type Si NWs/*n*-type ZnO film photodiode.

There have been several attempts to enhance the photocurrent efficiency of photodetectors by introducing new architectures based on transistors [11,12]. The established concept of the monolithic integrated system constructed with optoelectronic and electronic devices has prompted the idea that the transferred Si NWs can be used as the building blocks for a monolithic integrated system, because their length is sufficient to fabricate two functional devices on them. Thus, in this study, we propose a laterally coupled device composed of a *pn* heterojunction photodiode and a FET with a channel made of *p*-type Si NWs transferred onto a plastic substrate. Herein, the photodiode consists of the transferred *p*-type Si NWs and a sputtered *n*-type ZnO film. The transferred Si NWs in the laterally coupled device act not only as the channel for the FET, but also as the *p*-type material for the *pn* heterojunction photodiode. Figure 1 presents the electronic circuit diagram (a), the cross-sectional schematic of our laterally coupled device (b) and schematic energy band diagram of the heterojunction under thermal equilibrium (c). The energy gaps (E_G_) of the ZnO film and the Si NWs are 3.4 and 1.1 eV, respectively, and the electron affinities of the ZnO film and the Si NWs are 4.19 and 4.05 eV, respectively. A *pn* heterojunction photodiode composed of the *p*-type Si NWs and the *n*-type ZnO film is type-II. In this study, we examine the individual characteristics of the two-unit devices in the monolithic integrated system, and then investigate the performance of the photodiode modulated by operating the FET.

## 2. Experimental Procedure

**Figure 2.**
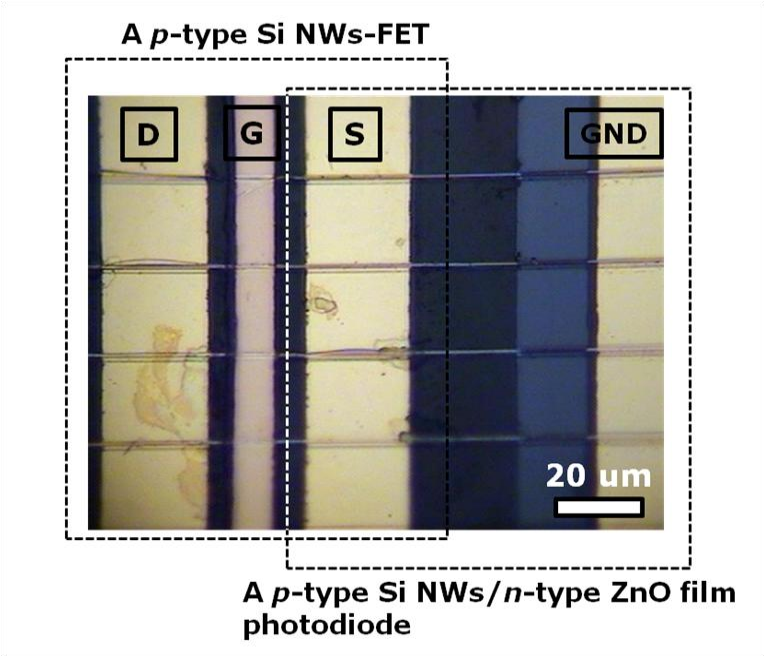
The optical top-view image of the laterally coupled device.

*P*-type Si NWs were obtained from a boron doped Si bulk wafer with a doping concentration of ∼10^17^ cm^−3^ using photolithography and an anisotropic wet etching processes [13]. The Si NWs with a diameter of 200 nm and a length of 300 μm were directly transferred onto a plastic substrate. The laterally coupled device was fabricated using the following procedures: (i) the pattern for a ZnO film was formed on one side of the transferred Si NWs by a photolithography process; (ii) the ZnO film was deposited on the pre-patterned region by rf magnetron sputtering [14] (note that the width, length, and thickness of the deposited ZnO film were 1 mm, 0.8 mm, and 180 nm, respectively, and that the sputtered ZnO film under study had clear *n*-type characteristics and good crystallinity (Figure S1, ESI†); (iii) Au electrodes with a thickness of 100 nm were deposited by thermal evaporation for use as the source and drain electrodes of the FET and the negative electrode of the photodiode; (iv) an Al_2_O_3_ layer with a thickness of 20 nm was formed by atomic layer deposition for use as the gate insulating layer of the FET; (v) finally an Au electrode was formed as the gate electrode of the FET. Figure 2 shows the optical top-view image of the laterally coupled device constructed with the photodiode and the FET. The electrical characteristics of the two single devices and the laterally coupled device were measured with a semiconductor parameter analyzer (Agilent 4155C) in air at room temperature. The excitation source for the photocurrent measurement was the 325-nm wavelength light from a He-Cd laser with a power density of 48 W/cm^2^ and neutral density filters were used to adjust the power of the incident light. The 325-nm wavelength light was irradiated onto the top-view side of the device. The illumination area was 28.3 mm^2^, and the entire area of the device was illuminated by the light since the beam size of the 325-nm wavelength light was much larger than the device size (8 mm^2^).

## 3. Results and Discussion

### 3.1. The Optoelectronic Characteristics of the Single Photodiode Composed of the Transferred p-type Si NWs and the n-type ZnO Film

**Figure 3.**
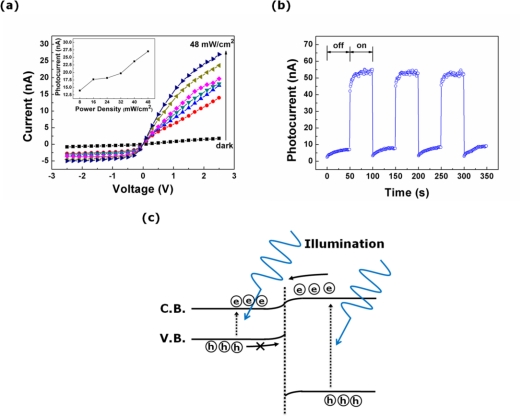
Optoelectronic characteristics of a *p*-type Si NW/*n*-type ZnO film photodiode. **(a)** The photocurrent characteristics under the illumination of the 325-nm wavelength light and the magnitude of the photocurrent depending on the power density of the light (inset); **(b)** the photocurrent transients under periodic illumination of light at a bias of 2.5 V; and **(c)** schematic energy band diagram in forward bias condition under the illumination of the 325-nm wavelength light.

Figure 3(a) shows the current-voltage (I–V) curves for the dark current and photocurrent for the single photodiode. The line shape of the I–V characteristics indicates the formation of a *pn* heterojunction between the transferred *p*-type Si NWs and the *n*-type ZnO film. The rectification ratio estimated at bias voltages of ±2.5 V increases from 2.3 in the dark to 5.5 under the illumination of light with a power density of 48 mW/cm^2^. The inset of Figure 3(a) shows the magnitude of the photocurrent as a function of the power density of the incident light at a bias voltage of 2.5 V. The magnitude of the photocurrent is proportional to the power density of the incident light. Note that the power density of the incident light increases from 8 to 48 mW/cm^2^ in intervals of 8 mW/cm^2^. When light with a power density of 48 mW/cm^2^ is illuminated onto the photodiode at a bias voltage of 2.5 V, the photocurrent efficiency and on/off ratio are determined to be about 2.3 μA/W and 14.4, respectively.

Figure 3(b) shows the photocurrent transient with a 50-second interval at a bias voltage of 2.5 V under the illumination of the 325-nm wavelength light with a power density of 48 mW/cm^2^. The photocurrent transient characteristics of our photodiode are similar to those reported in the studies of other research groups on *pn* heterojunction photodiodes composed of *n*-type ZnO and *p*-type Si [15,16], except for the phenomenon wherein the magnitude of the dark current grows slightly after the light is turned off. This phenomenon is attributed to the long length of our photodiode (The gap between the positive and negative electrode is estimated to be 40 μm). The photogenerated charge carriers adjacent to the electrodes rapidly move to the electrodes as soon as the light is turned off, resulting in initial fast photocurrent decay. On the other hand, the trapped charge carriers in the localized sites far away from electrodes are detrapped, and then drift toward the electrodes in the internal electric field. Consequently, this transport of the charge carriers is responsible for the increase of the dark current after the light is turned off. Figure 3(c) shows schematic energy band diagram of a *p*-type Si NWs/*n*-type ZnO film heterojunction photodiode in forward bias condition under the illumination of the 325-nm wavelength light. The difference (140 meV) between the electron affinities of the ZnO film and the Si NWs is much larger than the binding energy (60 meV) of excitons formed in the ZnO film. The large difference of the electron affinity enables the breaking of the excitons, and it can be the strong driving force for the charge separation. The photocurrent mechanism is briefly described, as follows; the detailed description on the forming and breaking of excitons and the charge separation are omitted here, for simplicity. The charge carriers are photogenerated in both regions of the Si NWs and the ZnO film when the light is irradiated on the device. Holes photogenerated in the Si NWs cannot pass through the junction potential barrier corresponding to the valence band offset, whereas electrons photogenerated in ZnO film transport toward the opposite site (or toward the positive electrode) in the internal electric field. Simultaneously, the photogenerated electrons in the Si NWs and holes in the ZnO film transport to positive and negative electrode, respectively. Consequently, the photogenerated charge carriers contribute to the photocurrent of the photodiode.

### 3.2. The Electrical Characteristics of the Transferred p-type Si NWs-FET

**Figure 4.**
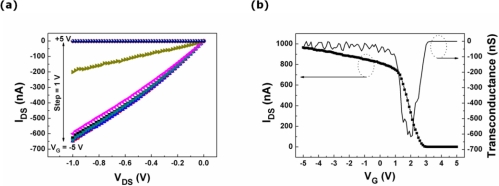
The electrical characteristics of the *p*-type Si NWs-FET. **(a)** The output characteristics and **(b)** the transfer characteristics (left axis) and the transconductance curve (right axis).

The drain-source current-voltage (I_DS_-V_DS_) curves taken from the *p*-type Si NWs-FET connected electrically to the photodiode in the laterally coupled device are illustrated in Figure 4(a) for gate voltage (V_G_) values ranging from +5 to −5 V with a step voltage of −1 V. The magnitude of I_DS_ increases as V_G_ decreases, indicating the *p*-type characteristics of the transferred Si NWs. Thus, this FET is able to be utilized as a switching component in the laterally coupled device. The transfer characteristics (left axis) and the transconductance (g_m_) curve (right axis) of the *p*-type Si NWs-FET at a V_DS_ of 1 V are illustrated in Figure 4(b). In the transfer characteristics, the I_on_/I_off_ ratio taken from the Si NWs-FET is estimated to be 2.6 × 10^5^, and the transconductance is found to be 617 nS at V_G_ = 2 V. The threshold voltage of the FET is evaluated from a linear fitting to the curve of the I_DS_ *versus* V_G_ with the extracted value of ∼2.5 V. The field-effect mobility taken from the *p*-type Si NWs-FET is calculated to be 21.9 cm^2^/V s at a V_DS_ of 1 V.

### 3.3. Characteristics of the Photodiode Modulated by the Operation of the FET in the Laterally Coupled Device

**Figure 5.**
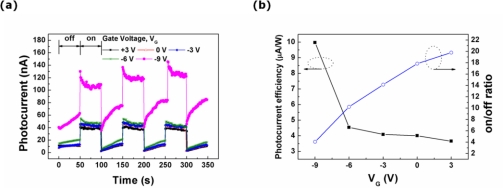
**(a)** The photocurrent transients of the photodiode coupled with the FET as a function of the gate voltage, and **(b)** the photocurrent efficiency and the on/off ratio as a function of the gate voltage.

The drain electrode of the FET is biased at a voltage (V_DD_) of 2.5 V in order to operate the laterally coupled device composed of the photodiode and the FET. Then, the characteristics of the photodiode are investigated for V_G_ values ranging from +3 to −9 V with a step voltage of −3 V. The photocurrent transients of the photodiode in the laterally coupled device are observed under the illumination of the 325-nm wavelength light with a 50-second interval as a function of V_G_, as presented in Figure 5(a). The magnitude of the dark current and photocurrent increases simultaneously as the value of V_G_ decreases. Figure 5(b) shows the dependence of the photocurrent efficiency and the on/off ratio on the gate voltage applied to the FET in the laterally coupled device. The photocurrent efficiency remains nearly constant as the gate voltage deceases from +3 to −6 V. The photocurrent efficiency of the photodiode coupled with the FET increases to 10.0 μA/W when the FET at a V_G_ of −9 V is switched on. On the other hand, the on/off ratio of the photodiode gradually decreases from 20 to 4 when the gate voltage is changed from +3 to −9 V. The dramatic increase in the magnitude of the dark current measured at a gate voltage of −9 V is responsible for the low on/off ratio (Figure S2, ESI†).

**Figure 6.**
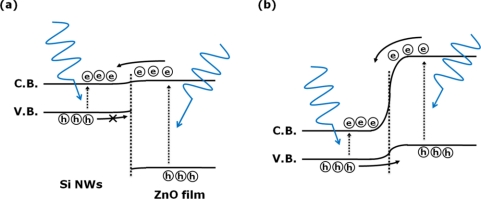
Schematic energy band diagrams of a *p*-type Si NWs/*n*-type ZnO film heterojunction photodiode coupled with a Si NWs-FET in forward bias condition under the illumination of the 325-nm wavelength light. The gate voltages of **(a)** +3 and **(b)** −9 V are applied on the gate electrode, respectively.

In comparison with the photocurrent transient of the single photodiode (as shown in Figure 3(b)), some differences ascribed to the potential barrier in the *pn* heterojunction are found in the characteristics of the photocurrent and the dark current. When a positive gate voltage of +3 V is applied, the conduction and valance bands in the Si NWs are raised up, which enlarges the height of the potential barrier for the photogenerated holes as demonstrated in Figure 6(a). This high potential barrier prevents the transport of the photogenerated holes toward the opposite electrode at positive gate voltages. Meanwhile, when a gate voltage of −9 V is applied, the photocurrent and the dark current considerably increase, due to the lowering of the height of the potential barrier between the valance bands of the ZnO film and the Si NWs as exhibited in Figure 6(b). Thus, the photogenerated holes move readily toward the negative electrode of the photodiode. The photocurrent efficiency of the photodiode coupled with the FET increases remarkably when the FET at V_G_ of −9 V is sufficiently switched on.

## 4. Conclusions

In this study, a laterally coupled device composed of a photodiode and a FET is fabricated on a plastic substrate by utilizing transferred Si NWs. The photodiode consists of transferred *p*-type Si NWs and a sputtered *n*-type ZnO film. The photocurrent efficiency of the photodiode coupled with a FET increases remarkably when the FET is switched on at a V_G_ of −9 V, and the value of 10.0 μA/W is five times larger than that of a single photodiode without any FET. Our study demonstrates that the photocurrent efficiency of a photodiode can be improved in a monolithic integrated system composed of a photodiode and FET and that the transferred Si NWs are an appropriate inorganic nanomaterial for the fabrication of a monolithic integrated system on a plastic substrate.

## Supplemental Information
