# Supplementary material for: Characterization of a Photodiode Coupled with a Si Nanowire-FET on a Plastic Substrate
Source: Sensors (Basel). 2010 Oct 12;10(10):9118–26. doi: 10.3390/s101009118 (PMC3230961; doi:10.3390/s101009118)

Supplemental Information

Figure S1. (a) The electrical characteristics (V_{DS}-I_{DS}) of the *n*-type ZnO film; and **(b)** the XRD pattern of the ZnO film deposited on a bare Si substrate. The V_{DS}-I_{DS} curves indicate that the sputtered ZnO film used in this study has clear *n*-type characteristics for the *pn* heterojunction photodiode. In addition, the crystallinity of the sputtered ZnO film is evaluated from the XRD pattern in a good agreement with the JCPDS card of ZnO (#75-1533). The good crystallinity of the sputtered ZnO film is observed from the XRD pattern in this study.

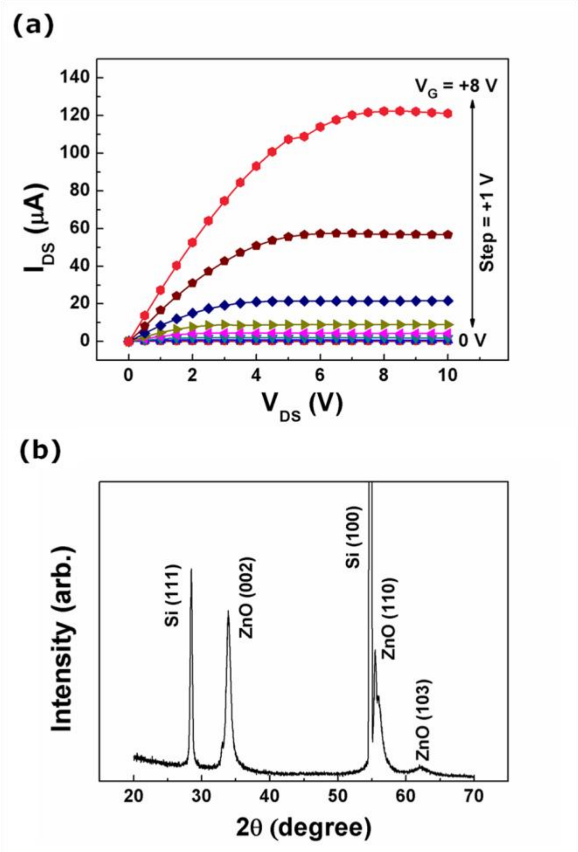

Figure S2. The magnitudes of the dark current and the photocurrent as a function of the gate voltage of the Si NWs-FET. When the gate voltage of −9 V is applied on the gate electrode, the magnitude of the dark current increases significantly, which is responsible for the low on/off ratio of the photodiode.

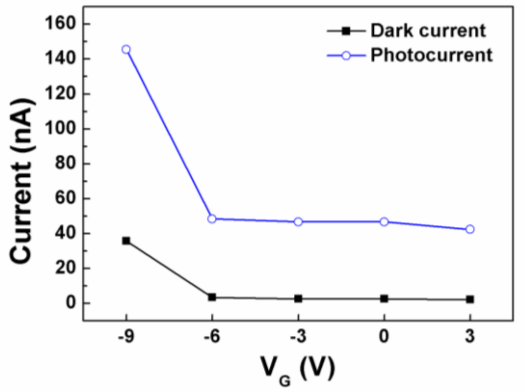

Supplement: Supplementary file 1 [file sensors-10-09118-s001.pdf]
